# Esophageal Schwannoma Presenting as Neck Mass and Misdiagnosed as Thyroid Nodule: A Case Report

**DOI:** 10.1002/oto2.32

**Published:** 2023-02-19

**Authors:** Nilnetre Mahathanaruk, Phurich Praneetvatakul

**Affiliations:** ^1^ Department of Otolaryngology‐Head and Neck Surgery, Faculty of Medicine Ramathibodi Hospital Bangkok Thailand

**Keywords:** esophageal tumor, neck mass, schwannoma

Benign esophageal tumors are rare, occurring at an incidence of <5%.^1^ Most are asymptomatic and incidentally found by imaging or endoscopy performed for other indications. Because esophageal carcinoma is approximately 50 times more common, the definitive diagnosis of a benign tumor is confirmed by pathology. We herein report a case of a benign esophageal tumor presenting as a palpable neck mass and mistakenly diagnosed as a thyroid nodule. The study was approved by the Ethics Committee of Ramathibodi Hospital, Mahidol University.

## Case Report

**Figure 1:**
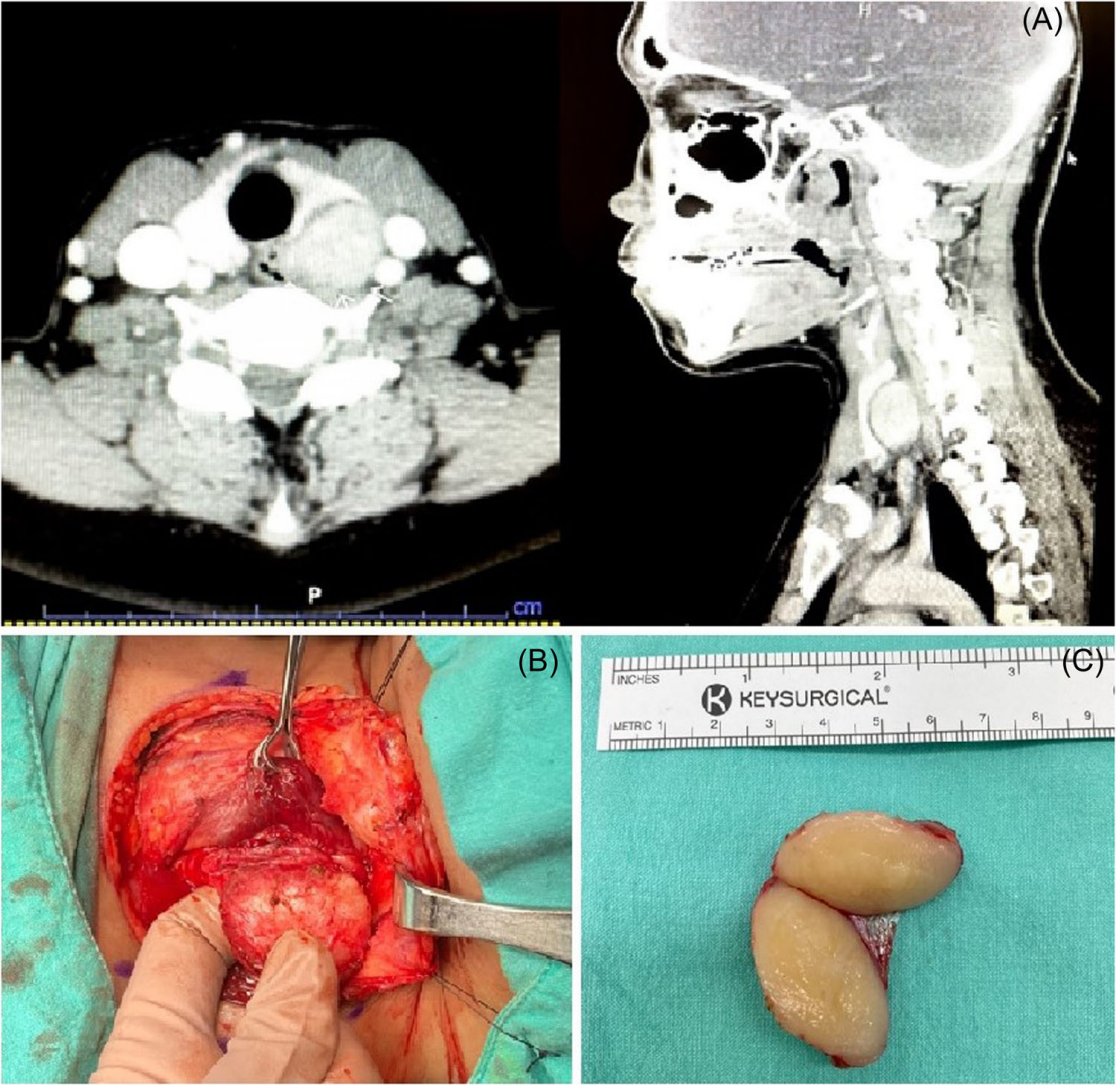
(A) Computed tomography scan showing the mass posterior to the left thyroid lobe with suspicion of esophageal wall involvement. (B) Recurrent laryngeal nerve after thyroid and tumor deflection. (C) Cut surface of the tumor showing an inhomogeneous white‐tan appearance.

A 36‐year‐old woman presented with a 1‐month history of an asymptomatic left neck mass. She had been previously diagnosed with a thyroid nodule. Her thyroid function test results were normal, and ultrasonography showed a well‐defined hypoechoic mass behind the left thyroid lobe. Computed tomography (CT) showed a 2.5‐ × 3.6‐cm enhanced mass at the left tracheoesophageal groove with suspicion of esophageal involvement (Figure 1A). Transnasal esophagoscopy (TNE) findings were normal. Fine‐needle aspiration biopsy revealed a spindle cell lesion. The patient was then scheduled for surgery.

**Figure 2:**
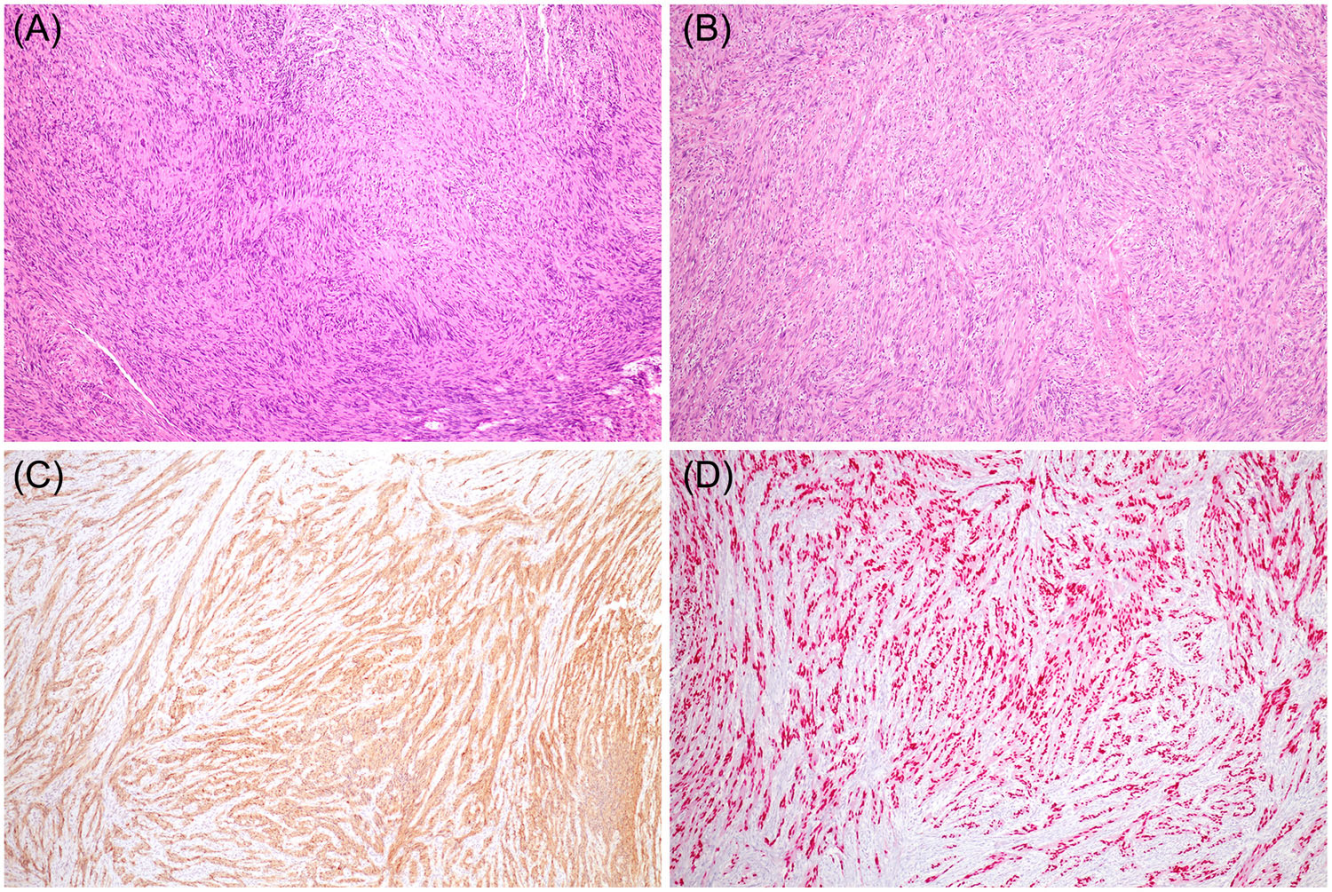
(A, B) Histopathologic examination of the tumor showed spindle cells with elongated wavy nuclei, a biphasic pattern, and focal nuclear palisading (hematoxylin and eosin, ×100). Immunohistochemistry showed positivity for (C) S‐100 (×100) and (D) SOX‐10 (×100).

A transcervical approach was performed. The tumor was found after deflection of the left thyroid lobe, and the recurrent laryngeal nerve was successfully preserved (Figure 1B). Intraoperative examination showed an encapsulated mass firmly attached to the esophagus. The tumor with its intact capsule was completely resected. The cut surface of the tumor showed an inhomogeneous tan‐white appearance (Figure 1C). The esophagus was repaired with a bipedicled sternohyoid muscle flap. A postoperative barium‐swallowing study showed no leakage. Histopathologic examination revealed a spindle cell tumor, highly suggestive of leiomyoma. Immunohistochemistry (IHC) was positive for smooth muscle actin, S‐100, SOX‐10, and caldesmon and negative for CD117, DOG1, CD34, and desmin; these findings were compatible with schwannoma (Figure 2). At the 1‐month follow‐up visit, the patient denied swallowing problems and other complications.

## Discussion

Benign esophageal tumors are considered rare compared with the higher incidence of esophageal carcinoma. In 2003, Choong and Meyers^2^ reported that the prevalence of benign esophageal tumors on autopsy was 0.17% to 0.59%, with the most common histological cell type being leiomyoma and the least common being schwannoma. Schwannoma is generally considered uncommon in the gastrointestinal tract, particularly in the esophagus, where <30 cases have been reported to date.^3^

The diagnosis relies on multiple modalities. In our case, physical examination led to an initial misdiagnosis of a thyroid lesion. Ultrasonographic findings provided more information regarding the esophagus, leading to more precise investigations (CT and TNE). Cytology was needed to rule out malignancy, and the result was clearly a spindle cell tumor. According to the 2005 World Health Organization oncopathology and genetics criteria, esophageal mesenchymal tumor can be diagnosed if spindle cells are found microscopically.^4^ With gastrointestinal stromal tumors (GISTs) and leiomyomas being the most commonly found, and considering the importance of differentiating the two because of the malignant potential of GISTs, tissue pathology with IHC is needed. Other diagnostic investigations usually consist of a swallowing study, esophagoscopy, endoscopic ultrasonography (EUS), CT, and positron emission tomography. EUS is popular as an initial investigation because it reveals the tumor characteristics without the risks associated with contrast agent. However, we did not perform EUS because the previous investigations provided adequate data in terms of tumor characteristics. Intraluminal evaluation was the only remaining procedure, and TNE, not EUS, could be readily performed in our setting at that time. The main treatment for benign esophageal tumor is surgery with different approaches (eg, video‐assisted thoracotomy, enucleation, or full‐thickness resection) depending on the tumor site and extent.

Our patient's initial pathological report suggested leiomyoma, but the histological pattern alone was not sufficient to differentiate other types of mesenchymal tumors; therefore, IHC was needed. Schwannomas are positive for S‐100 and SOX‐10 (characteristic markers for Schwann cells) and negative for CD117, CD34, and desmin. By contrast, GISTs are positive for CD117 and CD34, and leiomyomas are positive for actin, desmin, and caldesmon.^5^

The treatment options for esophageal schwannoma are based on many factors. Patients with asymptomatic schwannoma can undergo periodic surveillance with endoscopy, ultrasonography, or EUS. Most cases of symptomatic schwannoma warrant complete resection without further adjunctive treatment.

The prognosis of esophageal schwannoma is promising because of its very low recurrence rate after complete excision. However, if histopathology shows a high degree of mitosis, the prognosis might be worse in terms of malignant potential.

## Author Contributions

**Nilnetre Mahathanaruk**, surgeon, first author, corresponding author; **Phurich Praneetvatakul**, surgeon, coauthor, senior author.

## Disclosures

### Competing interests

None.

### Sponsorships

None.

### Funding source

None.
